# The T cell antigen receptor: the Swiss army knife of the immune system

**DOI:** 10.1111/cei.12622

**Published:** 2015-05-14

**Authors:** M Attaf, M Legut, D K Cole, A K Sewell

**Affiliations:** Division of Infection and Immunity, Cardiff University School of MedicineCardiff, UK

**Keywords:** CD1, MHC-I, MHC-II, MHC-Ib, MR1, T cell, T cell receptor, αβ TCR, γδ TCR

## Abstract

The mammalian T cell receptor (TCR) orchestrates immunity by responding to many billions of different ligands that it has never encountered before and cannot adapt to at the protein sequence level. This remarkable receptor exists in two main heterodimeric isoforms: αβ TCR and γδ TCR. The αβ TCR is expressed on the majority of peripheral T cells. Most αβ T cells recognize peptides, derived from degraded proteins, presented at the cell surface in molecular cradles called major histocompatibility complex (MHC) molecules. Recent reports have described other αβ T cell subsets. These ‘unconventional’ T cells bear TCRs that are capable of recognizing lipid ligands presented in the context of the MHC-like CD1 protein family or bacterial metabolites bound to the MHC-related protein 1 (MR1). γδ T cells constitute a minority of the T cell pool in human blood, but can represent up to half of total T cells in tissues such as the gut and skin. The identity of the preferred ligands for γδ T cells remains obscure, but it is now known that this receptor can also functionally engage CD1-lipid, or immunoglobulin (Ig) superfamily proteins called butyrophilins in the presence of pyrophosphate intermediates of bacterial lipid biosynthesis. Interactions between TCRs and these ligands allow the host to discriminate between self and non-self and co-ordinate an attack on the latter. Here, we describe how cells of the T lymphocyte lineage and their antigen receptors are generated and discuss the various modes of antigen recognition by these extraordinarily versatile receptors.

## Introduction

Immune surveillance by T lymphocytes is critical for the immune integrity of all jawed vertebrates and is imposed through an intricate armoury of functions that eliminate pathogen-infected and neoplastic cells. The T cell pool consists of several functionally and phenotypically heterogeneous subpopulations. T cells are broadly classified as αβ or γδ according to the somatically rearranged T cell receptor (TCR) they express at their surface.

αβ T cells are by far the most abundant and the best-characterized circulating T cells. Most αβ T cells recognize peptides from degraded proteins bound to major histocompatibility complex (MHC) molecules at the cell surface. These peptide-MHC (pMHC)-recognizing T cells were the first to be described 1. T cells that respond to pMHC are said to be ‘conventional’. However, a significant fraction of the αβ T cell pool consists of rarer T lymphocytes that do not recognize pMHC. These ‘unconventional’ αβ T cells include: (i) mucosal-associated invariant T (MAIT) cells that display limited diversity and are involved in anti-bacterial immunity 2,3; (ii) invariant natural killer T (iNK T) cells; and (iii) germline-encoded mycolyl**-**reactive (GEM) T cells. iNK T and GEM T cells are dedicated to recognition of glycolipids in the context of CD1d and CD1b, respectively 4,5. Other T cells are believed to recognize lipid antigens in the context of CD1a and CD1c, but these subsets have not been well characterized, or named, at the time of writing.

γδ T cells are also grouped as being ‘unconventional’, as they are not MHC-restricted and do not appear to recognize peptide antigens. One to 10% of circulating T cells express a γδ TCR, but this fraction is considerably higher in epithelial tissues. These tissues form the main portal of entry for pathogens, suggesting an important role of γδ T cells as early immune sentinels. Murine knock-out studies have demonstrated that γδ T cells have a clear role in pathogen clearance and tumour surveillance 6. Some T cells express a δ/αβ TCR hybrid. This is because the recombination mechanism used to generate TCR chains operates on the same locus for TCR-δ and TCR-α. Thus, some gene segments within the locus can, in some instances, be involved in the generation of TCR chains that are partially α and partially δ and are able to pair with canonical TCR-β chains 7. These so-called δ/αβ T cells constitute a significant proportion of T cells that recognize the CD1d-α-galactosylceramide (αGalCer) complex 7. Other hybrid TCRs further blur traditional segregation into αβ and γδ TCRs, as VγJβ, VβJγ, VδJβ, VδJγ and VγJδ TCR chains have been described, with or without the inclusion of Dβ and Dδ segments. Many of these combinations follow the 12/23 rule of V–(D)–J recombination, are transcribed into full transcripts and translated into hybrid proteins 8. The antigens recognized by T cells bearing these non-canonical TCR chains remain unknown, although a subset of Vγ (Dβ) Jβ TCRs are reported to be MHC-restricted 9.

Despite the functional and phenotypical differences between subsets, all T cells arise from the same precursors and share their early differentiation history. Thymic progenitor cells seed the thymus from the fetal liver or adult bone marrow. In the thymus, these progenitors enter a complex differentiation programme which leads to irreversible acquisition of T cell identity and expression of clonally distributed, variegated TCRs. In this review, we describe the mechanisms through which MHC-restricted and MHC-independent TCRs are generated and discuss recognition of antigen by distinct T cell subsets. We also re-examine recent findings on the more ‘unconventional’ subsets.

### Generation of diversity and thymic selection

**Figure 1:**
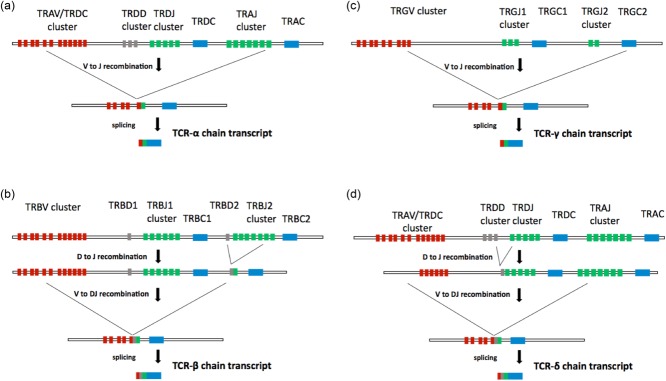
Generation of αβ and γδ T cell receptors (TCRs) by V–(D)–J recombination. (a) The *tra/trd* locus consists of a cluster of 46 functional T cell receptor alpha variable (TRAV) segments and eight T cell receptor delta variable (TRDV) segments, followed by three segments in the T cell receptor delta diversity (TRDD) cluster and four segments in the T cell receptor delta joining (TRDJ) cluster. A total of 51 functional TRAV segments lie between the TRDC and the T cell receptor alpha chain constant region (TRAC) segments. At the *tra/trd* locus, V–J recombination brings together one of many TRAV segments and one of many TRAJ segments. The intervening sequences are spliced out, producing a TCR-α transcript in which V, J and C segments are directly adjacent. (b) The *trb* locus comprises 48 functional T cell receptor beta variable (TRBV) segments followed by two D segments, 12 functional TRBJ segments and two TRBC segments. For TCR-β chain rearrangements, V–(D)–J recombination is a two-step, ordered process. D–J recombination occurs first, juxtaposing TRBD1 to one of many TRBJ1 segments or TRBD2 to one of many TRBJ2 segments. V–DJ recombination subsequently brings the rearranged DJ join to one of many TRBV segments. The intervening sequences are then spliced out, generating a TCR-β transcript in which V, D, J and C segments are directly adjacent. (c) The *trg* locus is composed of six functional TRGV segments and five TRGJ segments followed by two TRGC segments. At the *trg* locus, V–J recombination joins one of many TRGV segments to a TRGJ segment. Similar to TCR-α chains, the intervening regions are spliced, producing a TCR-γ transcript in which V, J and C segments are directly adjacent. (d) The generation of TCR-δ chains also occurs at the *tra/trd* locus. The 5′ end of this locus consists of a cluster of V genes. All V genes in this cluster can recombine with TRAJ, but only a subset can recombine with TRDD. Thus, many V genes in the *tra/trd* locus are used exclusively for TCR-δ rearrangement in early thymic precursors, while others are used exclusively for TCR-α rearrangement in double-positive thymocytes. Some V genes can be used interchangeably. Similar to TCR-β chains, TCR-δ chains are produced by V–(D)–J recombination and splicing, producing a final transcript in which V, D, J and C segments are directly juxtaposed. Unlike the TCR-β chain, however, TCR-δ can incorporate multiple D segments.

**Figure 2:**
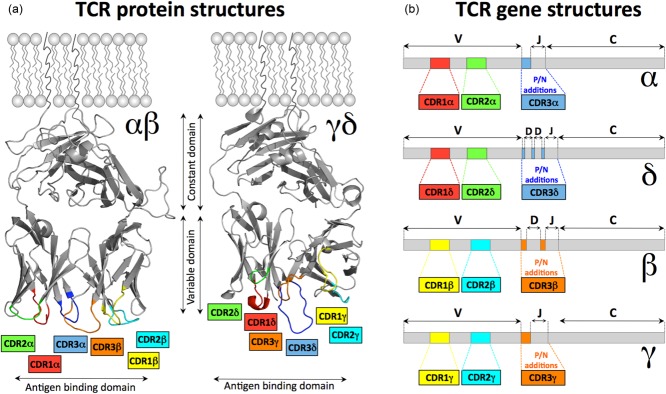
Structure of T cell receptor (TCR) proteins and mRNA. (a) αβ [Protein Data Bank (PDB): 3HG1] 10 and γδ (PDB: 1HXM) 11. TCRs adopt similar tertiary structures that position the complementarity-determining regions (CDR) loops at the membrane distal end of the molecules. Together the six CDR loops form the antigen binding site. (b) The mRNA structures show that for each chain CDR1 and CDR2 are encoded in the germline. CDR3 is the product of junctional diversity at V–J joins of T cell receptor (TCR)-α and TCR-γ chains and V–D–J joins in TCR-β and TCR-δ chains. CDR3 is consequently hypervariable. The colour code adopted for the CDR loops is maintained throughout this paper. The areas coloured in grey represent the constant and variable domains of the TCRs (not including the hypervariable CDR loops).

TCR diversity is generated somatically by gene rearrangement, a process that allows a vast array of different receptors to be produced from a limited set of genes. At the TCR-α locus (*tra*), discrete variable (V) and junctional (J) gene segments are recombined and juxtaposed to a constant (C) segment (Fig. 1a). Recombination at the TCR-β locus (*trb*) is similar, but includes an additional diversity (D) segment and one of two C segments can be appended to the rearranged TCR-β chain (Fig. 1b). The variability of TCRs is confined predominantly to three short hairpin loops on each chain, called complementarity determining regions (CDR). Collectively, the six CDR loops sit at the membrane-distal end of the TCR extracellular domain to form the antigen-binding site (Fig. 2a). The variable CDR1 and 2 loops are encoded in the germline by the T cell receptor alpha variable (TRAV) and T cell receptor beta variable (TRBV) gene segments. By contrast, the hypervariable CDR3 loops are generated by random deletion and addition of template and non-template nucleotides at the junction between recombining V, (D) and J gene segments (Fig. 2b). Polymorphism in the *tr* loci could add yet further diversity to the potential TCR repertoire at the population level 12. Theoretically, gene rearrangement by V–(D)–J recombination alone can produce ∼10^18^ TCRs in humans 13 and ∼10^15^ TCRs in the mouse 14. γδ TCRs are also generated by V–(D)–J recombination (Fig. 1c,d). The TCR-δ chain is thought to be by far the most diverse TCR chain due to the inclusion of multiple D segments, which can be translated in any reading frame (Fig. 2b). Thus, the theoretical number of different γδ TCRs that could be produced is potentially much greater than for the αβ TCR.

The quasi-random process of generating αβ TCRs described above has the capacity to generate receptors that are inept at recognizing self-MHC molecules and receptors that could be autoreactive. Thymic selection ensures that only T cells bearing a TCR that recognizes self-peptides in the context of self-MHC receive a survival signal. The majority of thymocytes do not receive this signal. Cells that express TCRs that cannot recognize self-pMHC are unlikely to be useful for recognizing foreign peptides. These cells do not receive a survival signal through their TCR and are said to ‘die by neglect’. At the other extreme, thymocytes that bear TCRs that react strongly to self-pMHC have the capacity to be autoreactive and are culled through a process of negative selection. Together, positive and negative selection ensure that only those αβ T cells that are restricted to recognizing self-pMHC within a low affinity range can populate the periphery. Thus, the thymic environment allows the generation of a pool of αβ T cells that are self-restricted, but not self-reactive 15.

Much less is known about selection of other, MHC-independent, T cell subsets. Invariant (type I) NK T cells are selected on CD1d-expressing CD4^+^CD8^+^ double-positive thymocytes and acquire effector function before exiting the thymus 16–18. Selection of MAIT cells has been shown recently to require MR1 expression on double-positive thymocytes 19. Commitment to the γδ T cell fate is thought to be a TCR-dependent process whereby strong γδ TCR signals induce γδ commitment and weak pre-TCR signals, in the absence of γδ TCR signalling, instruct thymocytes to initiate *tra* rearrangement 20. This model mirrors classic positive selection via the αβ TCR and suggests that γδ T cells may also need to encounter a cognate ligand in the thymus. Indeed, CD73 is up-regulated as a result of γδ TCR activation in the thymus. As CD73 is expressed by the majority of peripheral γδ T cells, ligand recognition in the thymus appears to be a common occurrence during γδ T cell development 21. However, potential ligands for γδ T cells are largely unknown. Skint-1 is the only known ligand required for maturation of Vγ5^+^ dendritic epidermal γδ T cells in the mouse, although its role in selection remains controversial 22. Interestingly, a study of murine T10/T22-reactive γδ T cells has indicated that, in contrast to αβ T cells, ligand recognition by the γδ TCR imprints effector function on the γδ T cell pool, but not antigen specificity 23. Overall, γδ T cell selection is poorly understood and the identity of potential positive selection ligands for γδ TCRs continues to be a matter of debate.

### Conventional, pMHC-restricted T cells

The cardinal feature of αβ T cells is the recognition of peptides derived from self and foreign proteins in the context of self-MHC molecules. The *mhc* locus was first described more than half a century ago as the set of genes which determine the outcome of tissue transplantation in congenic mice. It is now clear that the products of the *mhc* have evolved to allow T cells to perform highly specific functions which are crucial to adaptive immunity and host defence as a whole. The *mhc* locus is the most gene-dense and the most polymorphic region known to date, with more than 12 000 different alleles already described in humans 24. Polymorphism at the *mhc* ensures diversity in peptide presentation at the population level.

**Figure 3:**
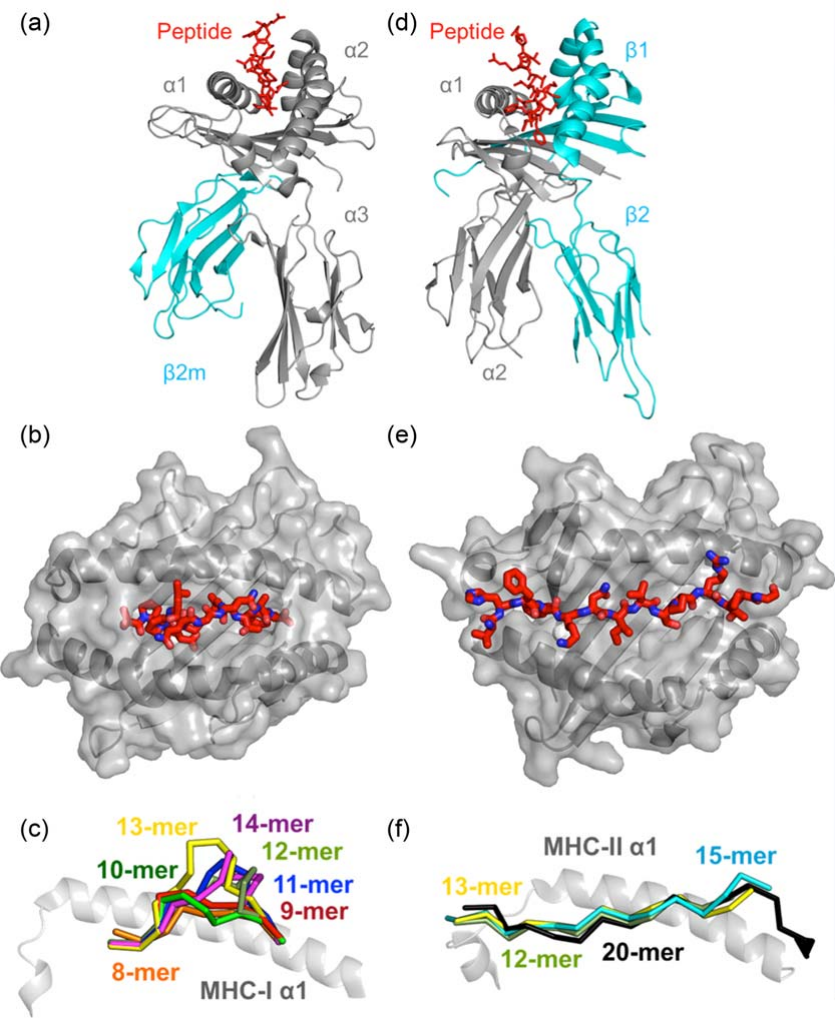
The structures of peptide-major histocompatibility complex class I (pMHC-I) and class II (pMHC-II). The two classes of classical MHC adopt similar overall structures despite being differently comprised. MHC-I [Protein Data Bank (PDB): 1ZHL] 25 (a) consists of a variable heavy chain (grey) folded with the invariant β-2-microglobulin molecule (cyan). (b) The ends of the MHC-I peptide-binding groove are closed. MHC-I presents peptides of 8–14 amino acids in length to T cells. (c) An 8-mer peptide can lie flat in the MHC-I groove. As additional amino acid residues are added, peptides have to bulge upwards and outwards in order to be accommodated within the groove. It has recently been established that MHC-I restricted T cells can recognize the length of a peptide in addition to its amino acid sequence (PDB: 1ZHL, 1XH3, 2FZ3, 3BW9, 1A1N, 1JF1, 1HHI) 25,27–33, whereas MHC-II (PDB: 1KG0) 26 (d) consists of an a-chain (grey) and a b-chain (cyan). Both MHC-I and MHC-II fold to present peptide (red) to T cells within a peptide-binding groove. (e) The open ends of the MHC-II peptide-binding groove allow presented peptides to extend at both the N- and C-terminus. Thus, MHC-II generally presents longer peptides than MHC-I. This mode of binding also means that the MHC-II can sometimes present the same peptide in different registers by using different amino acid side chains for anchoring into the MHC-binding pockets. (f) The open-ended MHC-II binding groove enables longer peptides to form an elongated conformation with peptide N- and C-terminal peptide flanking regions extending outside of the groove (PDB: 1KG0, 1UVQ, 2SEB, 2IAN) 26,34–36.

There are two types of classical MHC molecules: MHC class I (MHC-I) and MHC class II (MHC-II). There are three classical human MHC-I genes [human leucocyte antigen (HLA)-A, -B and -C]. These genes encode a membrane-spanning α-chain associated with the non-polymorphic β_2_ microglobulin (β_2_m) protein (Fig. 3a). Polymorphism is confined mainly to the membrane-distal α1 and α2 domains, while the α3 domain is largely invariant. The human MHC-II genes (DP, DQ and DR) encode two distinct polymorphic α and β-chains. Each chain folds into a membrane-distal polymorphic domain followed by a membrane-proximal immunoglobulin (Ig)-like domain (Fig. 3d) 37. The peptide-binding cleft of both MHC-I and MHC-II consists of two anti-parallel α-helices forming a channel in which the peptide can bind in an extended conformation on a platform of eight anti-parallel β-pleated sheets. Specific peptide-binding pockets in the base of this cleft vary between different MHC alleles 38. The binding affinity for MHC differs between peptides according to their primary sequence. A given peptide will, at best, bind a limited set of MHCs. Reciprocally, any MHC allele can only accommodate a small fraction of the peptide collection derived from a given protein.

### Conventional αβ TCR ligands

MHC-I molecules are expressed on nearly all nucleated cells and present peptides derived from endogenous proteins, allowing T cells to interrogate the internal proteome by scanning the surface of the target cell. MHC-II molecules differ from MHC-I in that they predominantly present peptides derived from exogenous proteins and are expressed primarily on professional antigen-presenting cells. Despite the similarities in overall conformation (Fig. 3), MHC-I and MHC-II present peptides in a distinct manner governed by the configuration of the peptide-binding cleft. Polymorphic residues define the size and chemical properties of the binding pockets within the cleft and therefore the peptide collection that can be accommodated by a given MHC-I molecule. The closed conformation of the MHC-I α_1_α_2_ binding cleft (Fig. 3b) restricts the length of peptides that can be accommodated. MHC-I molecules typically bind peptides of eight to 10 amino acids in length, but longer peptides have been observed in some instances 39. Because the MHC-I peptide-binding groove is closed at both ends, long peptides bulge out (Fig. 3c), exposing peptide side chains for direct interaction with the TCR 39. Curiously, our own recent studies have shown that MHC-I restricted TCRs appear to be predisposed to bind peptides of a defined length 27. In contrast, the MHC-II peptide-binding groove is an open-ended conformation (Fig. 3e) which allows the binding of N- and C-terminally extended peptides of up to 30 amino acids in length (Fig. 3f).

### TCR recognition of pMHC

**Figure 4:**
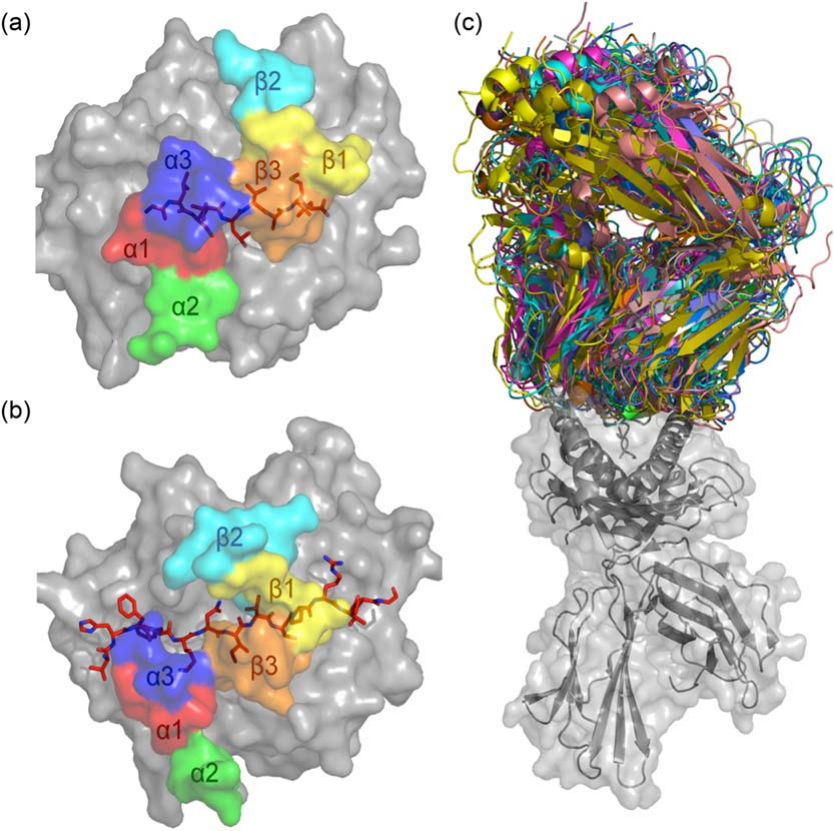
T cell receptor (TCR)–peptide-major histocompatibility complex (pMHC) structures. MHC molecules (in grey) and peptide (in red sticks) are overlaid with the docking footprints of the individual complementarity-determining regions (CDR) loops of the cognate αβTCR. The coloured footprints correspond to the colours of the CDR loops shown in Fig. 2. (a) Structure of MHC-I molecule HLA-A*0201 presenting the immunodominant GLCTLVAML peptide from Epstein–Barr virus (EBV) [Protein Data Bank (PDB): 3O4L] 40. The coloured footprint shows how the CDR loops of the AS01 TCR sit on the pMHC complex. This complex adopts a canonical conformation where the germline-encoded CDR1 and 2 loops contact mainly the MHC and the hypervariable CDR3 loops sit over the peptide. (b) Structure of the MS2-3C8TCR docked on the MHC class II molecule human leucocyte antigen (HLA)-DR4. Here, HLA-DR4 presents a peptide from myelin basic protein (PDB: 3O6F) 41. (c) Overlay of all MHC-I (grey cartoon and surface)-restricted TCRs (multi-coloured) in which co-complex structures have been solved. All complexes were aligned on the MHC-I molecule to demonstrate the flexible nature of TCR–pMHC binding.

MHC restriction is a defining characteristic of ‘conventional’ αβ T cells. Although MHC restriction was described more than 30 years ago, the molecular forces driving this interaction are still fiercely debated. Structural analyses of TCRs in their free and MHC-bound states have established that the TCR–pMHC interaction conforms to some ‘rules of engagement’. Indeed, most TCRs bind pMHC in a diagonal or nearly orthogonal orientation relative to the long axis of the MHC peptide-binding groove (Fig. 4a and b) 37. This conserved docking strategy, together with the observation that 20% of the pre-selection TCR repertoire is MHC-specific 42, has led to the idea that TCRs are genetically ‘hard-wired’ for MHC recognition 23–45. These rules fit conveniently with the observation that the germline-encoded CDR1α, CDR1β, CDR2α and CDR2β often contact the MHC α-helices, whereas the somatically rearranged CDR3α and CDR3β loops contact the peptide (Fig. 4c). This dogma was long supported by the identification of the so-called ‘interaction codons’ and the amino acids defining the MHC-I ‘restriction triad’ 44,46. More recently, however, Stadinski *et al*. 47 challenged the germline theory by demonstrating that the putative ‘interaction codons’ in a murine TCR-β chain were not strictly conserved, but were largely dependent on the identity of the partner TCR-α chain. Mutational studies on MHC alleles also suggest that TCR–pMHC interactions allow for substantial plasticity within the confines of a common binding and orientation system 48. A murine study in which CDR1 and CDR2 were randomized *in vivo* in the context of a fixed CDR3 failed to show preferential selection for the wild-type CDR1 and 2 sequences, contradicting the idea of TCRs being genetically ‘biased’ towards MHC ligands 49. It remains possible that the semi-conserved angle and polarity of TCR–pMHC interaction is required for the correct orientation of intracellular signalling domains, the positioning of the co-receptor binding site on MHC relative to the TCR or imposed by extracellular sites of glycosylation, as indicated by the crystal structure of a ternary TCR–pMHC–CD4 complex 50. Our recent studies show that TCR-peptide specificity overrides affinity-enhancing TCR–pMHC interactions to suggest that TCR CDR3 loops might need to ‘accept’ peptide prior to TCR engagement the MHC component of the ligand 51. Further work is required in order to dissect the sequence of molecular events involved in TCR–pMHC binding.

**Figure 5:**
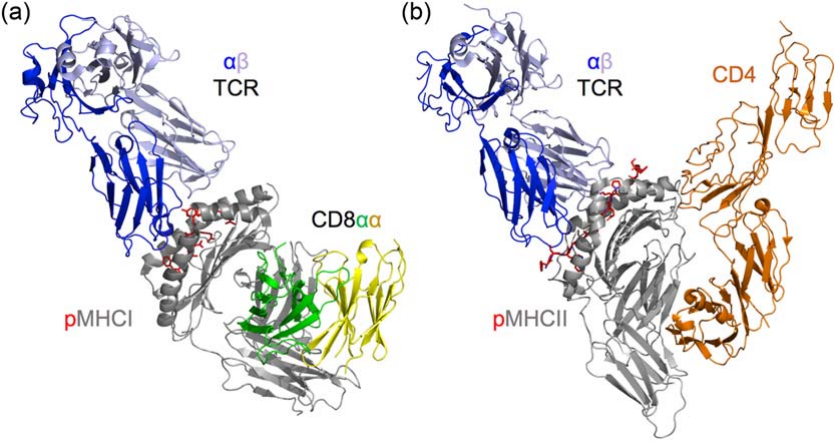
CD8 and CD4 co-receptors bind to invariant parts of peptide-major histocompatibility complex class I (pMHC-I) and pMHC-II, respectively. (a) Structural model of a T cell receptor (TCR)–pMHC-I–CD8 tripartite interaction [Protein Data Bank (PDB): 3O4L and 1AKJ] 40,52. MHC-I (grey cartoon) forms the peptide binding cleft using its α1 and α2 domains. The membrane distal face of the molecule comprises the TCR (blue and slate cartoon) docking platform. CD8 (green and yellow cartoon) binds at a distinct site on the α3 domain of the molecule. The structure shown is human leucocyte antigen (HLA) A*0201 and a CD8αα homodimer 52. (b) Tripartite complex structure of the TCR–pMHC-II–CD4 interaction (CD4 shown in orange cartoon) (PDB: 3T0E) 50. Similar to the TCR–pMHC-I–CD8 model, the CD4 co-receptor binds to an invariant site distal from the TCR binding platform.

An alternative to the germline theory of MHC restriction suggests that recognition of MHC-restricted ligands is the result of a selection process through which the peripheral repertoire is enriched with MHC-specific TCRs and purged of MHC-independent TCRs. In a series of studies involving MHC-I, MHC-II and co-receptor deficient-mice (referred to as ‘quad-deficient’ mice), it was shown that the selection of MHC-specific TCRs was strictly governed by the T cell co-receptors CD4 and CD8. CD4 and CD8 act as antigen co-receptors by binding to invariant regions of the MHC-II and MHC-I, respectively, at sites distinct from the TCR docking platform (Fig. 5). The cytoplasmic tail of the co-receptor binds to the protein tyrosine kinase Lck, which mediates key membrane-proximal phosphorylation events during T cell activation and selection. Singer and colleagues 53 first showed that the deletion of both co-receptors allowed selection of CD4^+^ and CD8^+^ T cells to take place on an MHC-deficient background. The authors worked on the premise that co-receptor-independent signalling can occur and is initiated by non-MHC ligand binding to the TCR, akin to cross-linking antibodies. By contrast, in the presence of co-receptors, which sequester all the available Lck needed for signalling, TCR signalling can be triggered only by MHC ligands. Thus, in the ‘quad-deficient’ mouse, non-MHC ligands can induce thymic selection of MHC-independent T cells – T cells which would otherwise die by neglect in a normal thymus 54–56. Reports of MHC-independent ligands for αβ T cells are scarce in the literature, although a number of examples have been observed in the mouse 57 and in humans 58,59. The existence of MHC-independent αβ TCRs in the periphery further supports the notion that MHC restriction is a TCR-extrinsic feature imposed on developing thymocytes through thymic selection.

### αβ TCR cross-reactivity

**Figure 6:**
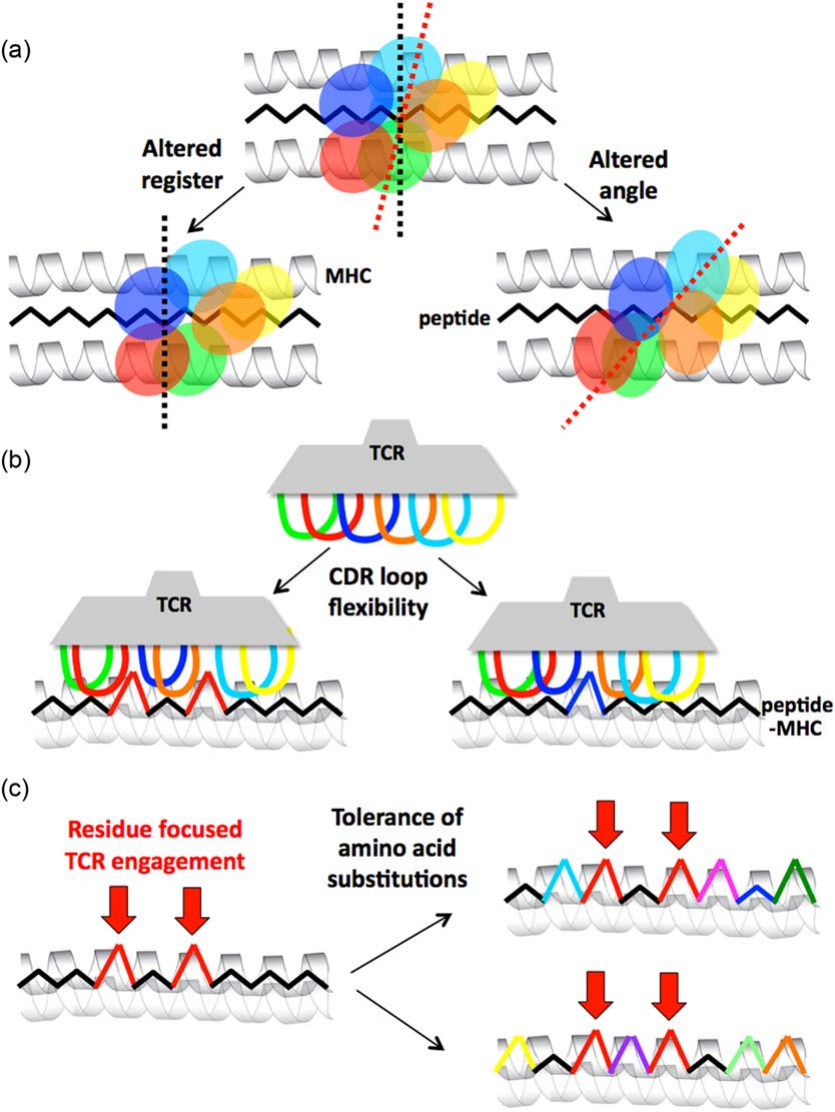
The plasticity of αβ T cell receptor (TCR) binding to peptide-major histocompatibility complex (pMHC). Individual TCRs use multiple mechanisms to bind to pMHC. These effects can increase the number of individual peptides that can be recognized. (a) Macro-level changes enable the TCR to bind pMHC with an altered angle or altered register. The cartoon shows the ‘footprints’ of TCR complementarity-determining region (CDR) loops projected down onto the pMHC. (b) Relatively micro-level flexibility in the CDR loops allows them to accommodate a variety of different shapes. The cartoon shows a side view of a TCR engaging pMHC. (c) The existing database of TCR–pMHC structures indicates that TCRs tend to focus interaction on two to four upward-facing amino acid residues in the antigenic peptide (so-called ‘hotspots’ 63). In this example a TCR might focus on two amino acids in the peptide (shown in red). Such residue-focused interaction then allows the TCR to accommodate multiple amino acid substitutions at other positions in the peptide (indicated by the use of different colours on the right). The three mechanisms described above are not mutually exclusive and represent just some of the possibilities. Many residues can also bind in individual MHC binding pockets. It is now understood that altering a primary MHC anchor can substantially change the way that a peptide might be viewed by incoming T cells 64,65.

**Figure 7:**
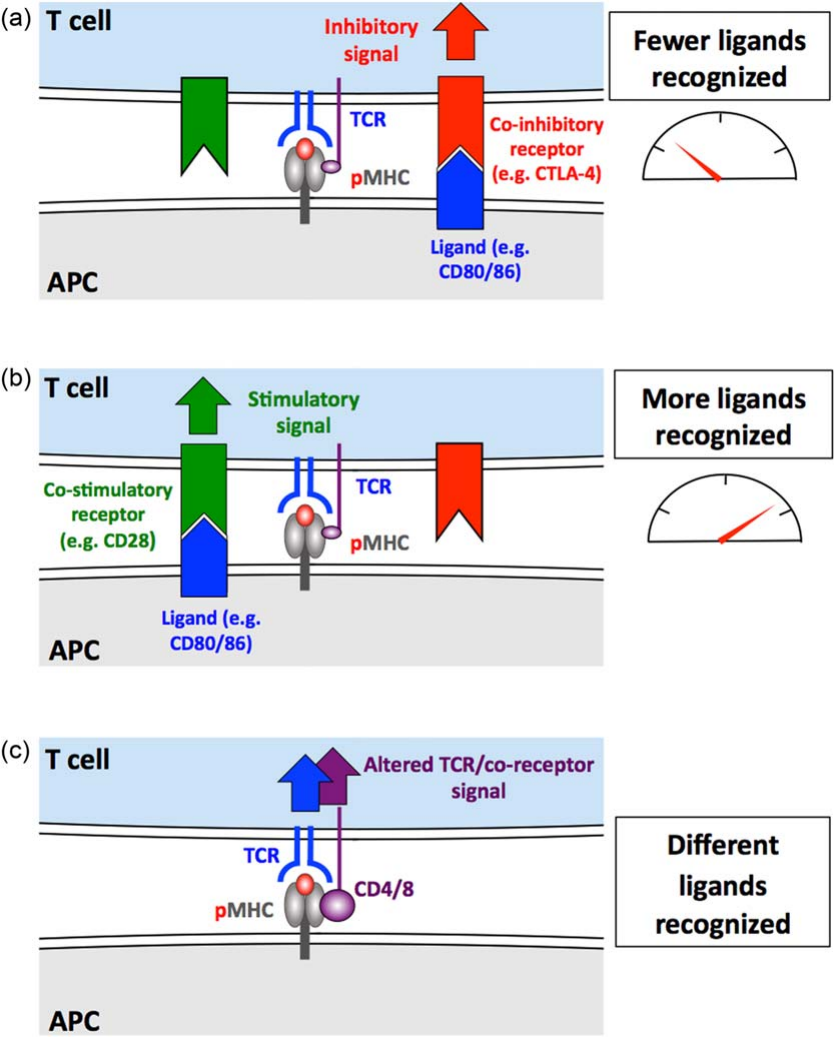
The peptide cross-reactivity of conventional T cells can be varied. In order to be positively selected in the thymus, a T cell must bear a T cell receptor (TCR) that allows it to recognize – and respond to – self-peptide. It should not respond to this peptide thereafter. T cell cross-reactivity could be regulated throughout the lifetime of a T cell. (a) Co-inhibitory (in red) or (b) co-stimulatory signals (in green) are likely to decrease and increase the number of ligands that can be recognized by tuning the sensitivity of TCR engagement that a T cell responds to. (c) The co-receptors, CD4 and CD8 (in purple), represent a special class of co-stimulation as these receptors bind to the same peptide–major histocompatibility complex (pMHC) ligand at the TCR. This could allow the co-receptors to discriminate between different TCR–pMHC dwell times and thus tune a T cell to recognize only certain ligands 66. Regulation of the cell surface expression and/or glycosylation of key receptors might also be used to vary sensitivity to cross-reactive TCR ligands.

As described above, the *mhc* locus is an example of extreme polymorphism. The vast majority of polymorphic residues in MHC proteins cluster around the peptide-binding cleft to suggest that this diversity is upheld to expand the variety of peptides that can be displayed to the immune system 38. The TCR must recognize peptides presented by all these variants. Beyond this, effective T cell immunity requires the TCR repertoire to recognize virtually any foreign peptide that can bind to host MHC molecules as a failure to recognize all possible foreign peptides would leave ‘blind spots’ that could be exploited by pathogens 60. Unlike the B cell receptor, the protein sequence of the TCR is fixed and never undergoes affinity maturation, so the TCRs expressed on existing naive T cells must be capable of responding to all alien antigens, despite never having encountered them before and being unable to adapt to them. The size of this task becomes apparent once it is realized just how many potential foreign peptides there are. This major evolutionary challenge has been met by enabling each TCR to interact with many – sometimes millions – of individual peptides bound in the groove of a single MHC 13,61. TCRs are further capable of engaging peptides presented by different foreign MHC alleles to promote alloreactivity. This enormous receptor plasticity is bestowed via a number of different mechanisms, as TCR binding can vary from being rigid and very focused 62 to being flexible in terms of both binding register and individual CDR loops (Fig. 6). It is also possible that the ability of T cells to respond to different TCR ligands could be varied during T cell development. TCR engagement in the presence of inhibitory signals is likely to dampen the number of ligands, whereas co-stimulatory signals would be expected to generate a greater level of T cell sensitivity and might increase the number of ligands that could be recognized (Fig. 7a,b). The co-receptors, CD8 and CD4, fall into a class of their own by co-engaging the pMHC ligand, thereby generating capacity for the molecules to alter which TCR ligands a T cell can respond to 67,66 (Fig. 7c).

### Implications of T cell cross-reactivity

**Figure 8:**
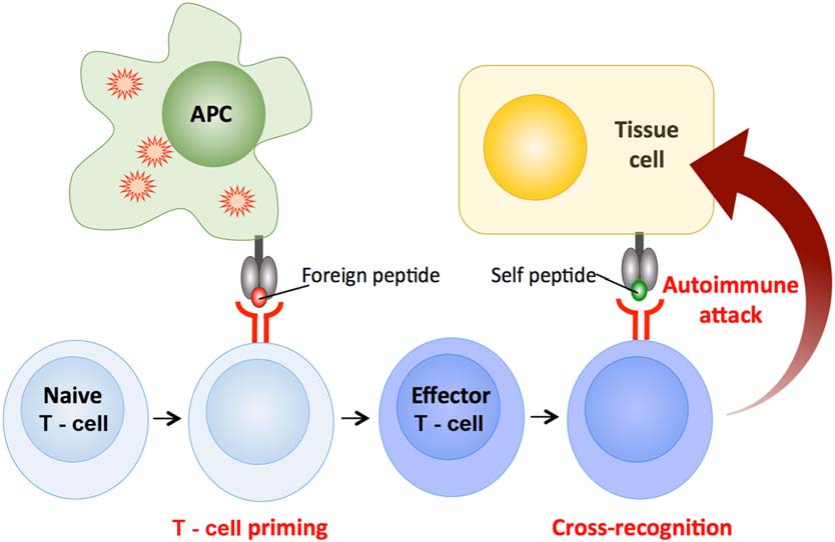
T cell cross-reactivity causes autoimmunity. T cells bearing autoreactive T cell receptors (TCRs) sometimes escape from thymic culling and populate the peripheral tissues. Such cells usually bear TCRs that bind very weakly to self-peptide and generally remain harmless. However, if such a T cell becomes activated in response to a pathogen-derived peptide it will be stimulated to become an effector T cell. Antigen-experienced cells are known to be more sensitive to TCR triggering. Activation of such cells by a cross-recognized self-peptide could then result in autoimmune attack.

It is becoming apparent that effective immune cover requires that each TCR must allow the T cell that bears it to respond to a large array of different peptide ligands 60. This extensive receptor cross-reactivity has a number of important consequences. First, it allows a relatively small number of TCRs (∼25 million 68) to provide effective immune cover for a vastly greater number of theoretical foreign peptides that could be encountered. Extensive T cell cross-reactivity also ensures that far fewer T cells are required to scan a cell displaying a foreign peptide before one reacts to this peptide, thus ensuring a more rapid response time. The corollary of far-reaching TCR cross-reactivity is that each peptide will be recognized by several different receptors (i.e. T cell responses are polyclonal). Escape from a polyclonal T cell response represents a far greater challenge for pathogens that can vary their antigens, as a mutation that escapes from one TCR may still be recognized by a different TCR. Heterologous immunity, where a single T cell can respond to several infections, is a further important consequence of individual TCRs being capable of responding to multiple peptides 69. Once it is realized that T cells can respond to many different peptides it should come as little surprise that there are pre-existing populations of HIV-specific memory T cells in people who are uninfected with this virus 70. The extent and implications of T cell heterologous immunity are described in detail elsewhere 13,69. The advantages of having a broadly cross-reactive T cell repertoire must outweigh the negatives, or the system would have been expected to evolve differently. Nevertheless, it is believed that widely cross-reactive T cells can have detrimental consequences. Most notably, the concept of T cells becoming activated by pathogens and then cross-recognizing self-ligands – a phenomenon known as molecular mimicry 71 – is believed to be the root cause of autoimmune disease (Fig. 8). A further important consequence of TCR binding plasticity is alloreactive recognition of non-self-HLA presenting self-peptide because such recognition causes acute rejection of HLA-mismatched grafted organs 72. Alloreactive recognition of pMHC by the TCR represents the major barrier to organ transplantation. Most transplants are HLA mismatched and require that the recipient take lifelong immunosuppressive medication, with its associated expense and side effects.

### Therapeutic use of the αβ TCR

**Figure 9:**
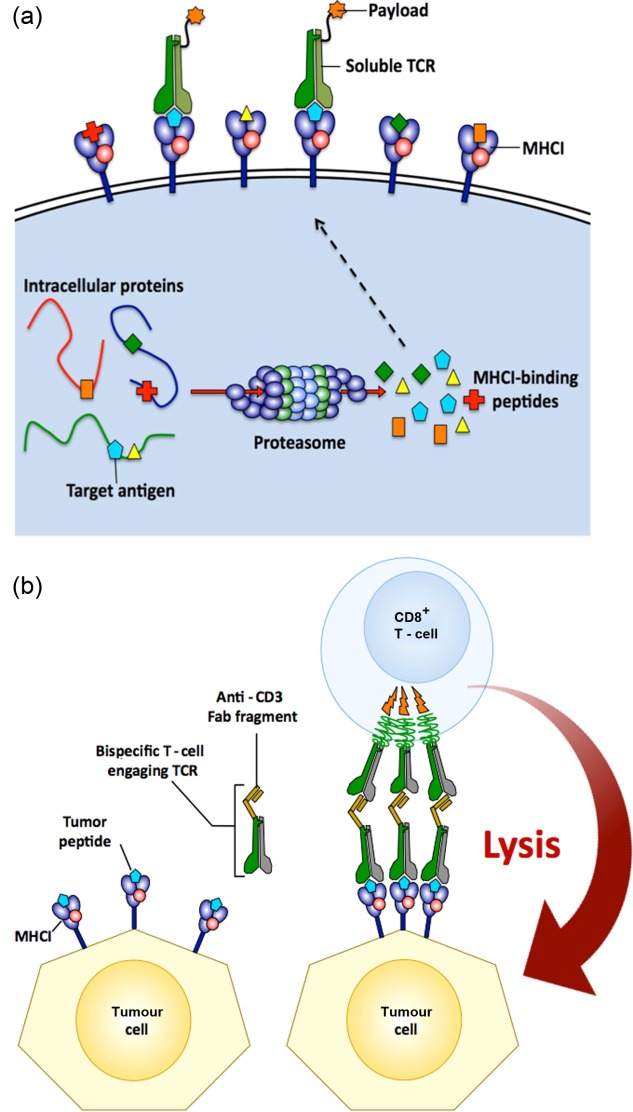
Soluble T cell receptor (TCR) therapy. (a) The MHC-I antigen presentation pathway is predicted to present at least one peptide from any protein present in a cell at 500 copies or more. This clever system allows the TCRs on the surface of MHC-I-restricted T cells to inspect the cellular proteome from the cell surface and detect internal anomalies. This ‘X-ray vision’ allows TCRs to access a far greater number of cellular targets than are available to monoclonal antibodies. Phage-display and directed evolution of TCRs 75 can generate very high-affinity molecules (K_D_ < 10 pM) that bind to the cognate peptide-MHC (pMHC) with very long half-lives (several hours). These molecules can then be used to deliver therapeutic payloads to specific cells *in vivo*. (b) High-affinity tumour-specific TCRs can be ‘fused’ to a CD3-specific Fab fragment to produce a bi-specific molecule. Such molecules have recently been used to induce cancer regression 86.

The transfer of TCR genes into recipient host T cells followed by the adoptive transfer of these cells to patients allows the passive transfer of immunity 73. This strategy can provide a convenient means for breaking tolerance to tumour antigens and has already shown promise in patients with malignant melanoma 74. As described above, T cell immunity is a compromise where a limited number of receptors are required to provide immune cover for a vastly greater number of potential foreign peptides bound to self-MHC. This compromise ensures that individual TCR–pMHC interactions are rarely optimal. Recent developments mean that TCRs can now be affinity-enhanced by phage display 75, yeast display 76 and computational design 77,78. Such enhancement has been used to build immune foresight by selecting TCRs that can recognize all known escape variants of HIV 79. Enhanced TCRs are particularly useful for cancer immunotherapy, as thymic selection culls T cells whose TCRs strongly recognize self-antigens. This process is presumably responsible for the finding that natural anti-tumour TCRs bind with substantially weaker affinity than anti-pathogen TCRs 80,81. This leaves cancer-specific T cells at a distinct disadvantage compared to their counterparts that respond to non-self-peptides. The enhancement processes described above can now be used to close the TCR binding affinity gap between optimal anti-pathogen TCRs and weaker anti-tumour TCRs. Enhanced optimal TCRs can then be used in TCR gene therapy for cancer 82. Such approaches are currently showing great promise. However, as enhanced TCRs have not undergone the rigours of thymic selection, where cells with TCRs that react strongly to self are deleted, they carry an inherent, but small, risk of being autoreactive. A recent trial of a TCR specific for the cancer-specific protein melanoma-associated antigen (MAGE) highlighted the potential problem. When cells with an enhanced MAGE-specific TCR were transfused back into two cancer patients, these patients developed rapid and fatal heart disease. Subsequent studies determined that the modified MAGE-specific TCR was also capable of cross-reacting with an MHC-I-presented peptide from the heart protein titin 83,84. Despite these teething problems, other trials with other receptors have been successful, and we anticipate that the use of such therapies will become commonplace in the next 20 years. Many groups are currently examining the use of various hybrid antigen receptors in T cell therapy. Such strategies are beyond the scope of this review, but have been documented recently elsewhere 85. Enhanced TCRs have also recently been used as soluble molecules to induce cancer regression 86, thus opening up a further exciting mechanism by which TCRs can be used for therapeutic benefit (Fig. 9). The use of TCRs as soluble ‘drugs’ does not incorporate the same dangers as cell-based TCR therapies, as dosage can be scaled or medication withdrawn if difficulties arise. The potential for commercial use of TCRs has been assessed elsewhere 87.

### Unconventional T cells

The list of unconventional T cells that do not recognize pMHC ligands is growing steadily, further demonstrating the versatility of the TCR. Recent discoveries have highlighted the existence of αβ T cells that recognize non-peptide ligands. Approximately 10% of all αβ T cells are now thought to recognize lipid antigens. A further 10% of human αβ T cells appear to recognize bacterial metabolites.

### αβ TCR recognition of lipids

**Figure 10:**
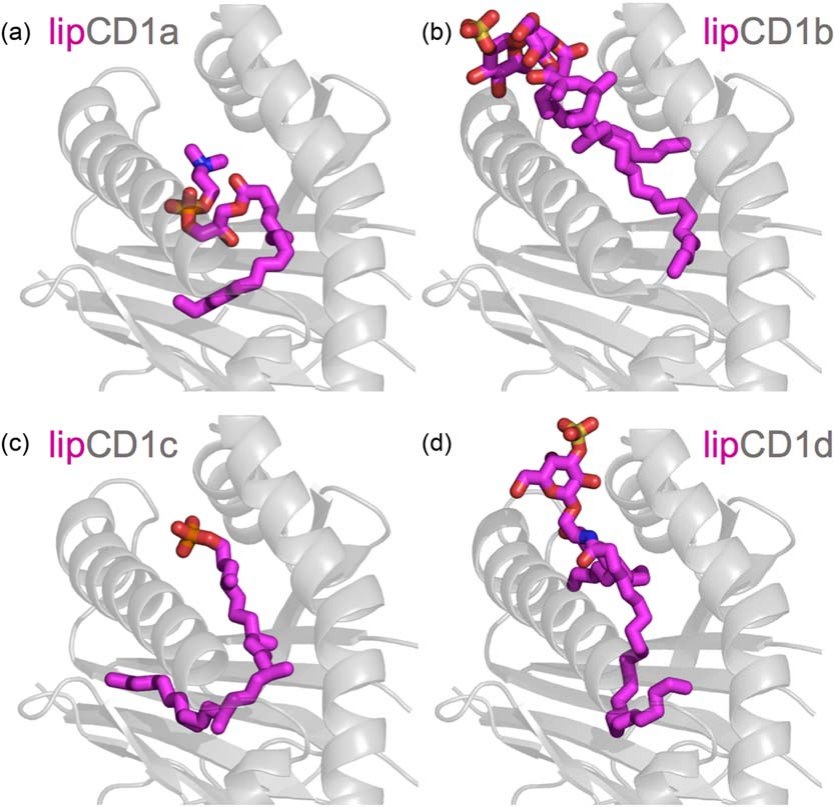
CD1a–d presentation of lipids. (a) CD1a can bind lipids not endowed with polar headgroups. Such lipids do not disrupt the T cell receptor (TCR)-activating conformation of the CD1a molecule. The presented ligand is lysophosphatidylcholine (LPC) [Protein Data Bank (PDB): 4X6E] 89. (b) CD1b can potentially present the largest and most diverse lipids of all CD1-family proteins. The lipid ligands can possess one, two or three alkyl chains buried in the hydrophobic pockets of CD1b while their polar headgroup can be recognized by germline-encoded mycolyl-reactive (GEM) T cells. The presented ligand is ganglioside GM2 (PDB: 1GZP) 90. (c) CD1c can present mycobacterial lipids such as phosphomycoketide (shown) to αβ T cells (PDB: 4ONO) 91. (d) CD1d can present self-derived lipids such as sulphatide (shown) or α-galactosylceramide (α-GalCer) to both αβ [type I and II natural killer (NK) T cells] and γδ T cells (PDB: 4MQ7) 92.

Many human αβ T cells have been identified recently that respond to non-peptide antigens presented by MHC class I-related molecules from the CD1 protein family 88. These glycoproteins are ideally suited for presentation of lipid-based antigens to T cells due to the hydrophobic nature of their deep antigen-binding pockets. There are five isoforms of CD1 molecules in humans, CD1a–e, although CD1e is not involved in antigen presentation. The antigen-binding clefts of CD1a–d differ in shape and size permitting the presentation of different lipids to T cells (Fig.10). T cells restricted by the group 1 CD1 molecules (CD1a–c) are considerably more numeric than CD1d-restricted T cells, but far less is known about these T cells and the TCRs that they express. At the time of writing, structures of group 1 CD1-restricted TCRs in complex with their antigen are only just starting to emerge. It is expected that further structures will appear soon in the literature.

**Figure 11:**
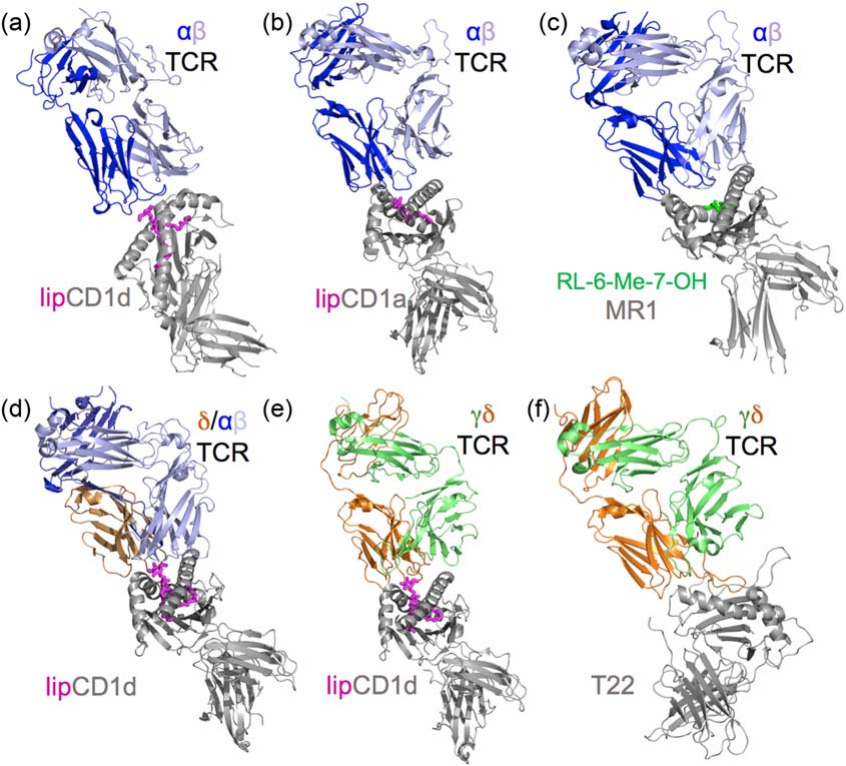
Recognition of antigen by ‘unconventional’ T cell receptors (TCRs). (a) Type I natural killer (NK) TCR recognition. The TCR binds CD1d–α-galactosylceramide (αGalCer) in a parallel docking mode. The recognition is markedly different from any known αβ TCR–peptide-MHC (pMHC) interactions [Protein Data Bank (PDB): 3VWK] 94. (b) Recognition of CD1a-lipid complex. αβ TCR recognizes a permissive conformation of CD1a surface that depends on the nature of the lipid ligand bound by the latter. Contrary to TCR-CD1d structures, no direct interactions between TCR and a lipid ligand bound to CD1a have been observed (PDB: 4X6C) 89. (c) Mucosal-associated invariant T (MAIT) TCR recognition of MR1. MR1 can present both activating (riboflavin precursors) and non-activating (folic acid precursors) intermediates from vitamin B biosynthetic pathways. The recognition of MR1 is mediated by conserved, invariant residues in an innate-like manner. The activating metabolites directly contact the TCR while no such interactions are observed for the non-activating metabolites (PDB: 4L4V) 95. (d) A hybrid δ/αβ TCR binds the CD1d-αGalCer complex. The TCR is composed of a variable Vδ1 domain fused with joining and constant α domains, and paired to a TCR-β chain. TCR-CD1d interactions are driven mainly by the germline-encoded Vδ1 residues while TCR-β mediates specific lipid recognition (PDB: 4WO4) 7. (e) The γδ TCR recognizes CD1d–lipid complexes. Germline-encoded Vδ1 residues are responsible for CD1d binding while the lipid ligand recognition is fine-tuned by hypervariable complementarity-determining region (CDR) 3 loops (PDB: 4MNG) 92. (f) A mouse γδ TCR binds the MHC-like molecule T22. The binding occurs predominantly via a conserved motif within the hypervariable CDR3δ loop, whereas the non-conserved CDR3δ residues fine-tune the affinity of the receptor towards T22 (PDB: 1YPZ) 96.

CD1d–lipid complexes bind to TCRs that are expressed by the innate-like NK T cells 93. CD1d-restricted T cells are divided into two types based on their TCR gene usage and the antigens to which they respond. Human type I NK T cells respond strongly to the lipid α-GalCer. These T cells typically utilize a semi-invariant TCR, and have been termed iNK T cells to reflect this. The type I NK T TCR uses an invariant TCR-α chain (TRAV10/TRAJ18) paired with a TRBV25.1-encoded β chain. Type II NK T cells express a broader TCR repertoire and are not activated by α-GalCer. NK T TCR recognition appears to be far more rigid than that observed for conventional recognition of pMHC. Type I NK T TCRs adopts a common footprint on CD1d (Fig. 11a). This recognition has been compared to an innate pattern recognition receptor. Recent type II NK T TCR–CD1d–sulphatide and lysosulphatide complexes have revealed that the mode of type II NK T TCR recognition is different to type I NK T TCR recognition 97. Type II NK T cells dock orthogonally and over the A′-pocket of CD1d and make a larger binding interface than type I NK T TCRs. The binding mode of type II NK T TCRs is closer to that used by conventional TCR docking on pMHC.

GEM T cells recognize mycobacteria-derived (glyco)lipids in the context of CD1b, a molecule which has the potential to accommodate the largest and most diverse lipid ligands among the CD1 protein family 90. Although no ternary structure of a TCR bound to the CD1b–lipid complex has been resolved so far, a recent study suggested that binding of the lipid antigen to CD1b could induce a conformational change of the latter. Thus, the TCR recognition would be mediated not only by the solvent-exposed polar headgroup of the lipid, but also by the rearranged residues from the antigen-presenting molecule 90. CD1c is also capable of presenting mycobacterial lipids and lipopeptides 98. Similarly to CD1b, TCR recognition of CD1c seems to be mediated by both the lipid antigen and CD1c residues; however, without showing a common pattern recognised by all TCRs responding to the same antigen 91. Interestingly, CD1c can also present immunogenic self-derived lipids accumulated in leukaemia cells, contributing to αβ T cell-mediated tumour surveillance 99.

Contrary to CD1b–d, CD1a can bind lipids lacking a polar headgroup. These hydrophobic lipid antigens are then not recognized directly by the TCR, but rather allow CD1a to adapt to a TCR-activating conformation 89 (Fig. 11b). In contrast, lipids containing a polar headgroup can also be bound by CD1a but disrupt the activating conformation of the antigen-presenting molecule, thus abolishing TCR-mediated recognition.

### MR1-restricted αβ TCRs

It has been shown recently that MAIT cells recognize intermediates in the riboflavin biosynthesis pathway bound to the MHC-like molecule MR1 100–102. MAIT cells constitute about 10% of all T cells in humans, so recognition is likely to represent the most dominant of all T cell antigen-specificities. Vitamin B is synthesized by some bacteria and yeast but not by mammals. Thus, such microbial-specific biosynthetic pathways provide a further mechanism that T cells can use to distinguish self from non-self. MAIT TCRs bind to riboflavin precursors and the presenting MR1 molecule (Fig. 11c). Interestingly, MR1 can also present non-activating metabolites belonging to the vitamin B group, namely folic acid derivatives. The invariant TCR-α chain binds MR1 in a conserved, innate-like manner, thus allowing the TCR to interact directly with the activating riboflavin precursor, while no such contact is made if the MR1-bound ligand is a folic acid derivative 95,103. MAIT cells have set a new paradigm in T cell recognition. As yet it is unclear how many other bacterial metabolites can be recognized by T cells, as a recent study by Corbett *et al*. 104 showed that MR1 is capable of capturing and presenting unstable intermediates of riboflavin synthesis, which would otherwise be converted into non-activating metabolites. However, it is established that the dominant subset of γδ T cells in human peripheral blood recognize intermediates in the non-mevalonate microbial pathway of isoprenoid biosynthesis, so it remains possible that a sizable fraction of human T cells also target other microbial-specific synthetic pathways.

### γδ TCRs

Surprisingly little is known about human γδ T cells or the ligands they recognize. Unlike αβ T cells, γδ T cells exhibit strict tissue-specific localization, which is determined by the identity of the rearranged TCR. This is because gene rearrangement at the *trg* and *trd* loci is programmed developmentally and different γδ T cells carrying distinct TCR-γ and TCR-δ rearrangements arise in successive waves that colonize different tissues during embryonic development 105. The tissue specificity of γδ T cells may also reflect a difference in the expression of γδ TCR ligands. Should this prove true, then γδ TCRs and their ligands might become very useful for tissue targeting. It remains to be seen whether γδ TCRs can ever bind to pMHC ligands. Nevertheless, structural studies of the complex between a murine γδ TCR and an MHC class I-like molecule T22 (Fig. 11f) indicate that at least some γδ TCRs can bind their ligand in a manner reminiscent of αβ TCR–MHC interactions 96,106.

Most progress with γδ TCRs has been made with the predominant subset of γδ T cells found in human peripheral blood that express a TCR made from the Vγ9 and Vδ2 genes. Vγ9^+^Vδ2^+^ T cells have been shown to recognize small pyrophosphate intermediates of the lipid biosynthetic pathway used by some bacteria 107,108. Notably, pyrophosphate antigens can accumulate in neoplastic cells as well, as a result of metabolic deregulation 109. While the structure of a Vγ9Vδ2 TCR has been solved 110, there is not yet a structure of this TCR bound to its ligand. Recognition of pyrophosphate ligands requires cellular presentation by a primate cell 111. This species specificity suggested that pyrophosphate antigens might be presented to Vγ9^+^Vδ2^+^ T cells by an MHC-like molecule. Recently the candidate for a pyrophosphate antigen-presenting molecule has been narrowed down to butyrophilin 3A1 (BTN3A1), an immunoglobulin-like molecule encoded in the MHC I locus. Crystallographic studies revealed that pyrophosphate antigens can be bound either in a shallow extracellular pocket of BTN3A1 and presented directly to T cells 112 or in an intracellular domain, enforcing a TCR-activating conformational change of the presenting molecule 113. These mechanisms, however, are not necessarily mutually exclusive, as both intra- and extracellular binding of phosphoantigens may be required for efficient TCR recognition.

**Figure 12:**
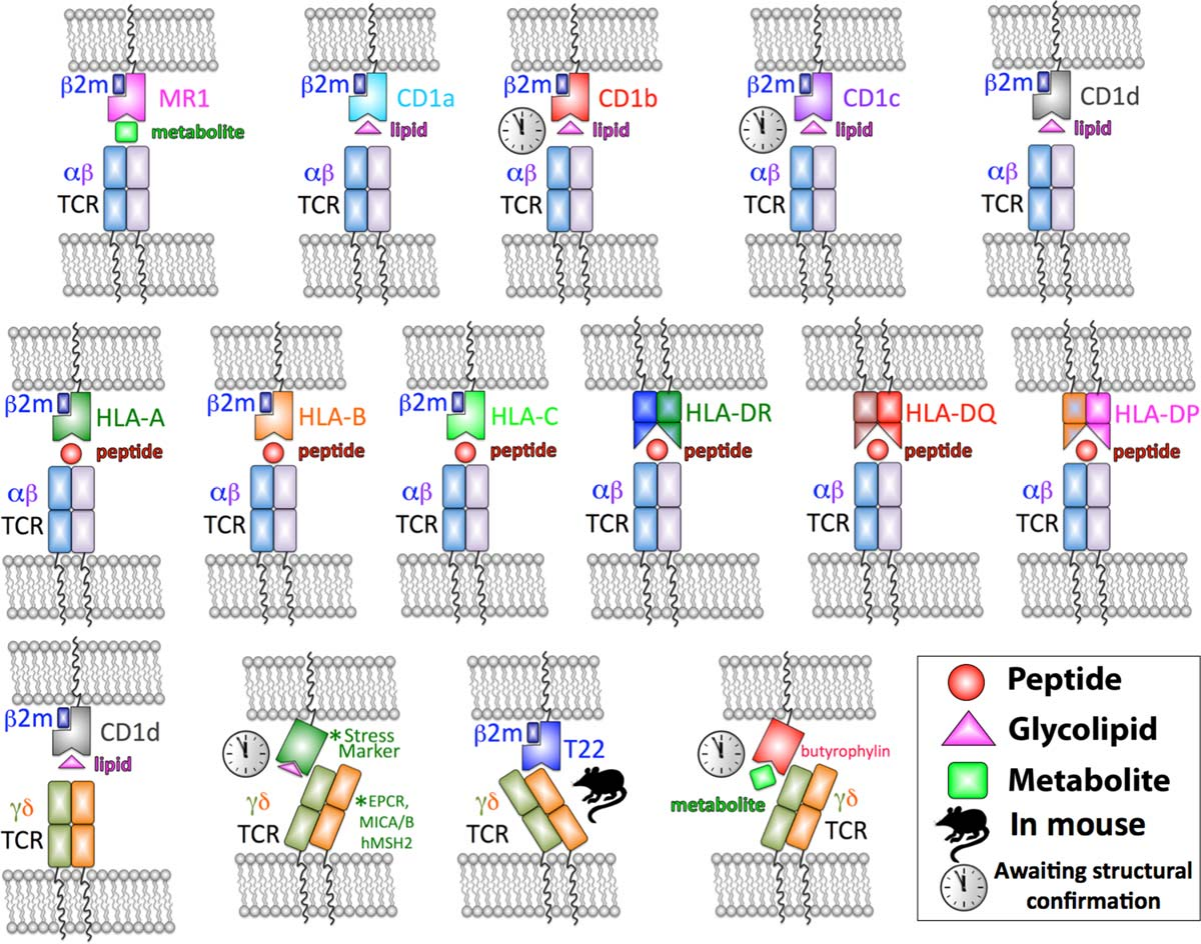
The versatility of the human T cell receptor (TCR). The αβ TCR can engage intermediates in riboflavin biosynthesis in the context of MR1 and a variety of lipid molecules bound to CD1a, CD1b, CD1c and CD1d. Classically, this receptor is also capable of binding to the almost infinite array of different peptides bound to more than 12 000 human leucocyte antigen (HLA) alleles of the HLA-A, -B, -C, -DR, -DQ and -DP loci. γδ TCRs can also bind to CD1d-lipid complexes 103,108 and a variety of ectopically expressed cell stress markers including endothelial protein C receptor (EPCR) 107 (shown), MIC A/B 114 and hMSH2 115. Murine γδ TCRs bind to the MHC-I like molecules T10 and T22 that are not found in humans 96. The best-studied set of human γδ T cells express Vγ9Vδ2 TCRs and recognize pyrophosphate antigens in the context of an immunoglobulin-like molecule, butyrophillin 3A1 112. The exact mechanism by which these TCRs recognize phosphoantigens awaits a TCR-ligand structure.

Similarly to ‘unconventional’ αβ TCRs, γδ TCRs can mediate recognition of lipids in the context of CD1d 92. However, the docking manner differed substantially from type I and II NK T cells (Fig. 11e). Importantly, the contacts with CD1d were mediated mainly via germline-encoded residues in CDR1 and 2 loops, while hypervariable CDR3 loops bound the presented lipid. Therefore, it is possible that γδ TCR can distinguish between subtly different lipid cargoes bound to CD1d in an adaptive-like manner. Additionally, Willcox *et al*. 107 have demonstrated recently that γδ TCRs can bind a ligand shared by cytomegalovirus-infected and malignantly transformed cells, namely endothelial protein C receptor (EPCR). EPRC shows homology with CD1 protein family at the level of amino acid sequence and structure, and is also capable of presenting lipids – however, the binding of the TCR to EPCR was lipid-independent (Fig. 12). The recognition was mediated via the hypervariable CDR3γ loop of the TCR, thus suggesting that EPCR binding was a part of an adaptive response rather than innate-like pattern recognition. Notably, EPCR was essential, but not sufficient, for the recognition of the target cells, highlighting the need for additional accessory molecules that would create a generalized cellular stress context. The identity of these accessory molecules on the T cell or target cell remains largely unknown.

EPCR is not the only example of a γδ TCR ligand recognized in a cellular stress context. It has been known for some time that a portion of human γδ T cells can bind MHC class I polypeptide-related sequence (MIC) A 114, a stress-related ligand of natural killer cells, and human homologue of the bacterial MutS protein (hMSH2) 115, an element of the DNA repair system. Both molecules can be up-regulated and expressed ectopically upon DNA damage, oxidative stress, malignant transformation and Epstein–Barr virus infection 116–118. Importantly, both MIC A 119 and hMSH2 116 were dually recognized by the TCR and a natural killer receptor NKG2D. Biophysical studies indicated that the ligand (MIC A) was bound initially by NKG2D forming a transient complex, giving way to a more stable TCR–ligand complex 119. It is therefore possible that this sequential recognition forms a critical part of immune surveillance, allowing the T cells to detect signs of cellular stress rapidly which could, in turn, indicate virally infected or transformed cells. The requirement for a multi-component stress signature could also serve as a preventive measure against triggering autoimmune reactions – a matter of particular importance when one considers that most of the known γδ TCR ligands are essentially self-derived. In line with this fact, γδ T cells have been shown to contribute to autoimmune diseases such as myositis and type I diabetes, as they can recognize aminoacyl-tRNA synthetases 120 and an insulin-derived peptide 121, respectively. The role of γδ T cells in pathogenesis of autoimmune diseases has been reviewed recently elsewhere 122.

## Summary

It has been known for several decades that the vast majority of TCRs recognize MHC-bound peptides. Recent developments have highlighted the astonishing versatility of this receptor (Fig. 2). Indeed, it is now widely accepted that a substantial fraction of T cell pool is dedicated to the recognition of non-MHC ligands. This includes recognition of various foreign lipids and metabolic intermediates and stress-related proteins. However, in the case of γδ T cells, the identity of putative ligands remains largely unknown. Although monoclonal antibodies now account for almost half of the new drugs that come onto the market, several TCR-based therapeutic strategies are in development. Such strategies are likely to have a bright future, and further exciting advances are anticipated shortly.
